# Significant Association between Serum Interleukin-6 and *Helicobacter pylori* Antibody Levels among *H. pylori*-Positive Japanese Adults

**DOI:** 10.1155/2013/142358

**Published:** 2013-12-22

**Authors:** Hiroko Nakagawa, Takashi Tamura, Yoko Mitsuda, Yasuyuki Goto, Yoshikazu Kamiya, Takaaki Kondo, Kenji Wakai, Nobuyuki Hamajima

**Affiliations:** ^1^Department of Preventive Medicine, Nagoya University Graduate School of Medicine, 65 Tsurumai-cho, Showa-ku, Nagoya 466-8550, Japan; ^2^Department of Hematology and Oncology, Higashi Nagoya National Hospital, National Hospital Organization, Nagoya 465-8620, Japan; ^3^Department of Pathophysiological Laboratory Sciences, Nagoya University Graduate School of Medicine, Nagoya 461-8673, Japan; ^4^Department of Healthcare Administration, Nagoya University Graduate School of Medicine, Nagoya 466-8550, Japan

## Abstract

*Background*. Interleukin-6 (IL-6) is a multifunctional cytokine produced by many types of cells. Inflammation plays a key role in the pathogenesis of atherosclerosis that is an underlying cause of coronary heart disease (CHD). Since the 1990s, some studies have shown an association between *H. pylori* infection and CHD, which may be mediated by inflammation. Therefore, this study aimed to evaluate the association between serum anti-*H. pylori* IgG levels and serum IL-6 levels in *H. pylori*-infected adults. *Methods.* We enrolled 158 subjects who visited a clinic located in an urban area to be tested for *H. pylori* infection, using the ^13^C-urea breath test, and who were found to be infected and subsequently received eradication. *Results.* The geometric mean serum IL-6 level was 1.78 pg/mL for men, 1.57 pg/mL for women, and 1.64 pg/mL overall. Logarithms of serum IL-6 levels were positively correlated with logarithms of serum *H. pylori* IgG levels (*r* = 0.24, *P* = 0.002). In multiple linear regression analysis adjusting for sex and age, the serum IL-6 level was still significantly associated with the IgG level in all subjects (*β* = 0.18, *P* = 0.012). *Conclusion.* Higher *H. pylori* IgG levels were significantly associated with higher serum IL-6 levels among *H. pylori*-infected individuals.

## 1. Introduction

Interleukin-6 (IL-6) is a multifunctional cytokine that is produced by many different types of cells, including monocytes, lymphocytes, fibroblasts, endothelial cells, keratinocytes, mesangial cells, and endometrial cells [1, 2]. Inflammation plays a key role in the pathogenesis of atherosclerosis [3–5] and contributes to plaque instability and thrombosis [5]. Atherosclerosis is the main pathological mechanism of coronary artery disease [3–6]. Some studies have shown that elevated serum C-reactive protein (CRP) levels were associated with coronary heart disease (CHD) events [7–11]. CRP is the major acute-phase reactant in humans, which is derived mainly from hepatocytes in response to IL-6 [10]. In addition, some epidemiological investigations have shown a direct association between circulating IL-6 levels and risk or severity of CHD [11–15].

After the first report by Mendall et al. [16] in 1994, a considerable number of studies have been performed on the correlation between *Helicobacter pylori* (*H. pylori*) infection and CHD. Some studies have shown a positive association between them [16–22], while others have shown no significant association [23–27]; a hypothesis was proposed that *H. pylori* infection might play an important role in altering systemic inflammation levels that could be connected to CHD. However, only one study has investigated the association between *H. pylori* antibody titers and IL-6 levels [28], which reported a significant correlation between them in 107 healthy subjects [28]. To further elucidate the relationship between *H. pylori* infection and serum IL-6 levels, we conducted a cross-sectional study to examine the association between serum anti-*H. pylori* immunoglobulin G (IgG) levels and serum IL-6 levels in Japanese *H. pylori*-infected adults.

## 2. Methods

### 2.1. Subjects

Subjects were Japanese adults who visited an urban clinic in Nagoya, Japan, between December 2005 and October 2010, for the purpose of being tested for *H. pylori* infection and for subsequent eradication. They were apparently healthy individuals who were concerned about possible *H. pylori* infection. During this period, we enrolled 158 *H. pylori*-infected individuals (54 men and 104 women) aged 23–78 years. Subjects with gastric cancer or idiopathic thrombocytopenia or those not infected with *H. pylori* or who had an unknown infection (*n* = 109) were not included, nor were those with serum IL-6 >4 pg/mL (*n* = 12) or with no data of *H. pylori* antibody levels (*n* = 9). All subjects gave written informed consent to take part in this study, which was approved by the Ethics Committee of Nagoya University School of Medicine (approval number 155).

### 2.2. Clinical Tests

All participants provided blood samples. Serum IL-6 levels and pepsinogen (PG) isozymes I and II were measured using a CLEIA (chemiluminescence enzyme immunoassay). The reference range for serum IL-6 was ≤4 pg/mL for both sexes. Serological atrophic gastritis was defined as a serum PG I level of ≤70 ng/mL and a PG I/II ratio of ≤3.0. Subjects were classified into 3 groups according to serological levels of pepsinogens: strong positive (PG I level of ≤30 ng/mL and a PG I/II ratio of 2.0), moderate positive (PG I level of ≤50 ng/mL and a PG I/II ratio of 3.0), and positive (PG I level of ≤70 ng/mL and a PG I/II ratio of ≤3.0 but neither moderate nor strong positive) [29–33]. The rest of the subjects were regarded as serological atrophic gastritis negative. *H. pylori* infection was determined using the ^13^C-urea breath test, with positive results defined as a value 2.5‰, which is the cutoff value recommended in Japan. The serum anti-*H. pylori* IgG level was assessed using an EIA (enzyme immunoassay).

### 2.3. Statistical Analysis

Since the log_10_-transformed values of serum IL-6 levels and the log_10_-transformed values of serum *H. pylori* IgG levels showed a near-normal distribution, they were used in the analysis. Age was stratified in 10-year increments. The strength of association between the logarithm of serum IL-6 levels and the logarithm of serum anti-*H. pylori* IgG levels was examined using the Pearson correlation coefficient. Multivariate regression analysis was performed to assess the correlation of log_10_-transformed serum IL-6 with log_10_-transformed serum anti-*H. pylori* IgG levels with adjustment for sex and age; logarithm of serum anti-*H. pylori* IgG levels was incorporated as a dependent variable. Dummy variables indicating levels of atrophic gastritis were used in the regression analysis (no atrophic gastritis, positive, moderate positive, and strong positive). Additional multivariate regression analysis was performed to assess the influence of atrophic gastritis on log_10_-transformed serum anti-*H. pylori* IgG levels with adjustment for sex and age. A two-sided *P* value of less than 0.05 was considered statistically significant. All statistical analyses were performed using Stata version 11.1 software (STATA Corporation, College Station, TX, USA).

## 3. Results

Subject characteristics are shown in Table 1. The mean age was 56.9 years (58.4 years in men and 56.2 years in women) and most were aged 60–69 years for both sexes. The geometric mean of serum* H. pylori* IgG titer was 51.3 and the serum *H. pylori* IgG level ranged from 6 to 280. The geometric mean serum IL-6 level was 1.78 pg/mL for men, 1.57 pg/mL for women, and 1.64 pg/mL overall; the level ranged from 1.0 to 2.0 pg/mL among 53.7% of men and 58.7% of women.

**Figure 1:**
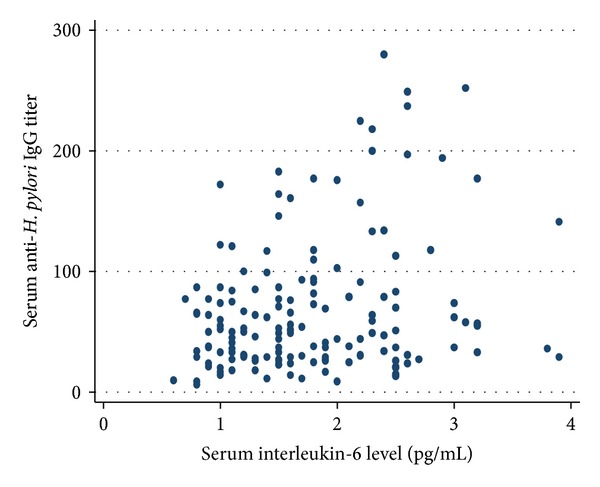
Correlation between serum interleukin-6 and anti-*H. pylori* IgG titers among men (*r* = 0.27, *P* = 0.045, and *n* = 54).

**Figure 2:**
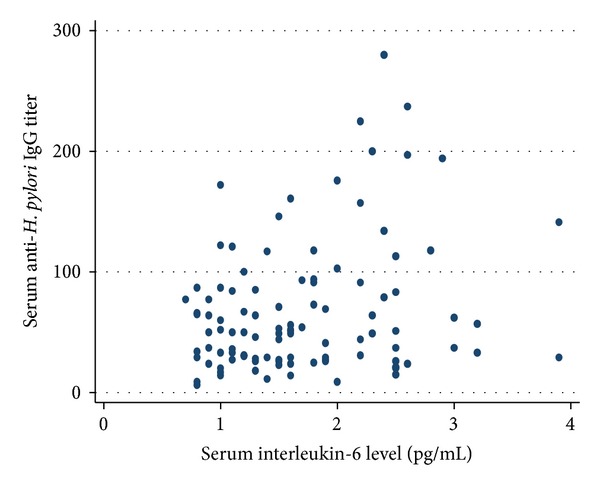
Correlation between serum interleukin-6 and anti-*H. pylori* IgG titers among women (*r* = 0.23, *P* = 0.021, and *n* = 104).

Figures 1 and 2 show the scatter plots of serum anti-*H. pylori* IgG levels against serum IL-6 levels in men and women, respectively. The logarithm of serum IL-6 levels was positively correlated with the logarithm of serum *H. pylori* IgG levels in men (*r* = 0.27, *P* = 0.0045), in women (*r* = 0.23, *P* = 0.021), and in both sexes combined (*r* = 0.24, *P* = 0.002). In multiple linear regression analysis, adjusting for sex and age, a higher log_10_-transformed serum IL-6 level was still significantly associated with a higher log_10_-transformed serum anti-*H. pylori* IgG level in all subjects (*β* = 0.18, *P* = 0.012). The association was similarly observed in both men and women, though it did not reach statistical significance in either sex; *β* = 0.20 and *P* = 0.11 for men and *β* = 0.17 and *P* = 0.055 for women. When a cut-off value of 5% for the ^13^C-urea breath test, which is used worldwide, was applied as an *H. pylori*-positive definition, the results were not modified in any of the subjects (*β* = 0.14, *P* = 0.045). When stratified by status of atrophic gastritis, the association was significant among those without serological atrophic gastritis (*β* = 0.26, *P* = 0.011) and not significant among those with positive (*β* = −0.046, *P* = 0.75), moderate positive (*β* = 0.20, *P* = 0.254), or strong positive (*β* = −0.22, *P* = 0.93) serological atrophic gastritis. After adjustment for sex and age, atrophic gastritis was not significantly associated with the logarithm of serum *H. pylori* IgG levels (*β* = −0.0011, *P* = 0.99 for positive, *β* = −0.074, *P* = 0.27 for moderate positive, and *β* = −0.12, *P* = 0.15 for strong positive). Even in the multiple linear regression analysis adjusting for sex, age, and atrophic gastritis, a higher log_10_-transformed serum IL-6 level was significantly associated with a higher log_10_-transformed serum anti-*H. pylori* IgG level in all subjects (*β* = 0.17,  *P* = 0.020).

## 4. Discussion

The present study showed that *H. pylori-*positive Japanese adults with a higher anti-*H. pylori *IgG titer had a higher concentration of IL-6. The association in this study is consistent with the result of a previous study that involved 107 Caucasians [28].

Over the years, a growing body of evidence has demonstrated that higher levels of inflammatory markers such as serum IL-6 and CRP were associated with an increased risk of developing CHD. One systematic review showed that circulating IL-6 was associated with an increased risk of cardiovascular disease [12]. In two prospective studies, evaluating associations with long-term average circulating IL-6 levels provided an odds ratio for CHD risk of 2.14 [13]. Recently, a prospective cohort study showed that a one-SD increment in log-transformed serum IL-6 was positively associated with an increased risk of cardiovascular mortality, with a hazard ratio (HR) of 2.04 (95% CI, 1.34–3.68) [14]. In the Fragmin and Fast Revascularization During Instability in Coronary Artery Disease II trial, elevated IL-6 (>5 ng/L) was associated with a higher 6- and 12-month mortality, independent of troponin or high-sensitivity CRP [15]. A large prospective case-cohort study showed that increased concentrations of CRP in men and IL-6 in women were strong and independent predictors of CHD risk even after adjustment for traditional cardiovascular risk factors [11]. CRP has also been shown to be induced in response to IL-6 [10]. A systematic review and meta-analysis showed that the summary estimate of relative risk for incident CHD was 1.58 (95% CI, 1.37–1.83) for CRP levels greater than 3.0 mg/l, compared with levels of less than 1.0 mg/L [9]. Although our subjects had serum IL-6 levels ≤ 4 pg/mL, which is within standard values, many cohort studies have shown a significant association between IL-6 levels and the risk of CHD when IL-6 levels were within standard values [11, 13]. A prospective case-cohort study showed an association between IL-6 concentrations and the incidence of CHD, even when IL-6 levels were within standard values (3.1 pg/mL in cases and 2.0 pg/mL in noncases; *P* < 0.001) [11]. Similarly, the risk of CHD by fifths of baseline circulating IL-6 levels in a pooled analysis of participants without known CHD at baseline in the Reykjavik Study and British Regional Heart Study has increased continuously with increasing fifths of circulating IL-6 levels, even when they are within standard values [13]. The association found between serum IL-6 levels and anti-*H. pylori* IgG levels in the current study could play an important role epidemiologically and clinically. Established risk factors, such as genetics, diet, and exercise, play a more important role than *H. pylori*. However, epidemiological studies have shown an association between CHD and *H. pylori* infection when these risk factors are taken into consideration. Moreover, a number of cohort studies have shown that IL-6 level is a risk factor of CHD, independent of these factors [11, 13]. Therefore, the association between IL-6 levels and anti-*H. pylori* antibody levels in this study is thought to be important.

Although controversial, a large number of studies have been published on the possible role of *H. pylori* infection in cardiovascular disease. A previous study showed that the prevalence of *H. pylori* infection was significantly higher in patients than in controls (62% versus 40%; *P* = 0.004), with an odds ratio of 2.8 (95% CI: 1.3–7.4; *P* < 0.001) adjusted for age, sex, main cardiovascular risk factors, and social class [19]. A case-control study on 1122 patients with acute myocardial infraction and 1122 age- and sex-matched controls showed a higher prevalence of *H. pylori* seropositivity in acute myocardial infraction patients than in controls, even after adjustment for socioeconomic status (OR: 1.87, 95% CI: 1.42–2.47, and *P* < 0.0001) [20].

Our findings suggest that a strong immune response to* H. pylori* enhanced the systemic inflammation, which was reflected in an increased level of serum IL-6. With regard to the biological mechanisms for this finding, previous studies have shown that HP0175, which is a secreted peptidyl prolyl cis-trans-isomerase of *H. pylori*, elicits IL-6 gene expression and IL-6 release from macrophages [34, 35]. A chronic low-grade inflammatory process leading to atherosclerosis could be one possible mechanism involved in the onset of *H. pylor*i-induced ischemic heart disease [36]. In fact, a study found that *H. pylori* IgG levels were higher for subjects who died of ischemic heart disease compared with those of survivors and higher for survivors compared with those of controls [37]. In addition, one study showed that patients with Henoch-Schönlein purpura had significantly higher levels of anti-*H. pylori* IgG compared with healthy controls (86 ± 32 versus 32.5 ± 23 U/mL) [38]. The anti-*H. pylori* IgG antibody titer may play a crucial role in other extradigestive diseases as well as cardiovascular disease.

It has been previously shown that serum anti-*H. pylori* IgG antibody titers were significantly correlated with the severity of inflammation in both the antrum and body of the stomach [38]. Significant associations were found between serum anti-*H. pylori* IgG and IgA antibody titers and the development of atrophic gastritis [39]. The IgG* H. pylori* antibody absorbance index was significantly correlated with not only the density of antral *H. pylori* colonization but also the degree of gastritis of the antrum, as assessed using the Whitehead score and activity, as well as the Sydney system [40]. In contrast to this study, our findings showed that atrophic gastritis was not significantly associated with serum *H. pylori* IgG levels after adjustment for sex and age. Furthermore, serum IL-6 levels were significantly associated with *H. pylori* IgG levels independent of sex, age, and atrophic gastritis stage. Thus, the possible elevation of serum IL-6 due to *H. pylori* infection was not determined by the level of atrophic gastritis.

In the current study, there was no significant difference in serum IL-6 levels between infected and uninfected subjects. In multiple linear regression analysis adjusted for sex and age, *H. pylori* infection status (uninfected = 0, infected = 1) was not significantly associated with IL-6 levels in all of the subjects (infected and uninfected) (*β* = 0.047, *P* = 0.411). Therefore, uninfected (*n* = 93) subjects and those with unknown infections (*n* = 16) were excluded from this study. This is a limitation of this study. However, we conducted this study to assess the correlation between serum anti-*H. pylori* IgG levels and serum IL-6 levels in *H. pylori*-infected subjects. Therefore, this study evaluated the association of serum IL-6 levels with the immune response to *H. pylori* infection as assessed by antibody levels.

## 5. Conclusions

In conclusion, our study revealed a significant positive association between serum IL-6 levels and anti-*H. pylori* IgG levels in *H. pylori*-infected Japanese adults. Because this may imply that the immune response to *H. pylori* promotes systemic inflammation, further studies are needed to confirm this association.

## Figures and Tables

**Table 1:** Distribution of age, serum anti-*H. pylori* IgG, interleukin-6 level, and atrophic gastritis by sex.

Characteristics	Men		Women		Total	
	*n*	%	*n*	%	*n*	%
Age (years)						
20–29	1	1.9	0	0.0	1	0.6
30–39	5	9.3	7	6.7	12	7.6
40–49	3	5.6	18	17.3	21	13.3
50–59	15	27.8	34	32.7	49	31.0
60–69	26	48.1	41	39.4	67	42.4
70–79	4	7.4	4	3.8	8	5.1
Serum anti-*H. pylori* IgG titer						
<50	26	48.1	49	47.1	75	47.5
50–100	19	35.2	35	33.7	54	34.2
101–200	6	11.1	17	16.3	23	14.6
>200	3	5.6	3	2.9	6	3.8
Serum interleukin-6 (pg/mL)						
<1.0	3	5.6	13	12.5	16	10.1
1.0-2.0	29	53.7	61	58.7	90	57.0
2.1–3.0	17	31.5	26	33.8	43	27.2
3.1–4.0	5	9.3	4	3.8	9	5.7
Atrophic gastritis						
Negative	21	38.9	40	38.5	61	38.6
Positive	11	20.4	20	19.2	31	19.6
Moderate positive	12	22.2	31	29.8	43	27.2
Strong positive	10	18.5	13	12.5	23	14.6

Total	54	100.0	104	100.0	158	100.0
